# Genome-Wide Investigation Reveals Potential Therapeutic Targets in *Shigella* spp.

**DOI:** 10.1155/2024/5554208

**Published:** 2024-03-21

**Authors:** Md. Arju Hossain, Md. Al Amin, Md. Arif Khan, Md. Rashedur Rahman Refat, Md Sohel, Md Habibur Rahman, Ariful Islam, M. Nazmul Hoque

**Affiliations:** ^1^Department of Biotechnology and Genetic Engineering, Mawlana Bhashani Science and Technology University, Tangail 1902, Bangladesh; ^2^Department of Microbiology, Primeasia University, Dhaka 1213, Bangladesh; ^3^Institute of Epidemiology, Disease Control and Research (IEDCR), Dhaka 1212, Bangladesh; ^4^EcoHealth Alliance, New York, NY 10018, USA; ^5^Department of Information and Communication Technology, Mawlana Bhashani Science and Technology University, Tangail 1902, Bangladesh; ^6^Department of Biochemistry and Molecular Biology, Primeasia University, Banani, Dhaka 1213, Bangladesh; ^7^Department of Biochemistry and Molecular Biology, Mawlana Bhashani Science and Technology University, Santosh, Tangail 1902, Bangladesh; ^8^Department of Computer Science and Engineering, Islamic University, Kushtia 7003, Bangladesh; ^9^Center for Advanced Bioinformatics and Artificial Intelligence Research, Islamic University, Kushtia 7003, Bangladesh; ^10^Department of Gynecology, Obstetrics and Reproductive Health, Bangabandhu Sheikh Mujibur Rahman Agricultural University, Gazipur 1706, Bangladesh

## Abstract

*Shigella* stands as a major contributor to bacterial dysentery worldwide *scale*, particularly in developing countries with inadequate sanitation and hygiene. The emergence of multidrug-resistant strains exacerbates the challenge of treating *Shigella* infections, particularly in regions where access to healthcare and alternative antibiotics is limited. Therefore, investigations on how bacteria evade antibiotics and eventually develop resistance could open new avenues for research to develop novel therapeutics. The aim of this study was to analyze whole genome sequence (WGS) of human pathogenic *Shigella* spp. to elucidate the antibiotic resistance genes (ARGs) and their mechanism of resistance, gene-drug interactions, protein-protein interactions, and functional pathways to screen potential therapeutic candidate(s). We comprehensively analyzed 45 WGS of *Shigella*, including *S. flexneri* (*n* = 17), *S. dysenteriae* (*n* = 14), *S. boydii* (*n* = 11), and *S. sonnei* (*n* = 13), through different bioinformatics tools. Evolutionary phylogenetic analysis showed three distinct clades among the circulating strains of *Shigella* worldwide, with less genomic diversity. In this study, 2,146 ARGs were predicted in 45 genomes (average 47.69 ARGs/genome), of which only 91 ARGs were found to be shared across the genomes. Majority of these ARGs conferred their resistance through antibiotic efflux pump (51.0%) followed by antibiotic target alteration (23%) and antibiotic target replacement (18%). We identified 13 hub proteins, of which four proteins (e.g., tolC, acrR, mdtA, and gyrA) were detected as potential hub proteins to be associated with antibiotic efflux pump and target alteration mechanisms. These hub proteins were significantly (*p* < 0.05) enriched in biological process, molecular function, and cellular components. Therefore, the finding of this study suggests that human pathogenic *Shigella* strains harbored a wide range of ARGs that confer resistance through antibiotic efflux pumps and antibiotic target modification mechanisms, which must be taken into account to devise and formulate treatment strategy against this pathogen. Moreover, the identified hub proteins could be exploited to design and develop novel therapeutics against MDR pathogens like *Shigella*.

## 1. Introduction

*Shigella* spp. are ubiquitous, nonmotile, non-spore-forming, rod-shaped Gram-negative bacteria that thrive in a wide range of conditions [1, 2]. *Shigella* spp. are the causative organism of shigellosis, which is an acute gastroenteritis infection [3, 4]. *Shigella* infections continue to be unacceptably high creating a global human health problem [4, 5]. According to the World Health Organization (WHO), each year shigellosis causes ~700 000 deaths worldwide [6]. The *Shigella* spp. consist of four species such as *S. dysenteriae* (serogroup A), *S. flexneri* (serogroup B), *S. boydii* (serogroup C), and *S. sonnei* (serogroup D) and >50 serotypes [7, 8]. A shift in the epidemiology of *Shigella* serogroups has been observed in recent years. *S. flexneri* is mostly prevalent in developing countries, whereas *S. sonnei* is mostly prevalent in developed countries [9]. *S. boydii* is most commonly isolated in Bangladesh, India, and other Southeast Asian countries, though *S. dysenteriae* (especially serotype 1) plays only a minor role in the endemicity of shigellosis in recent years [7, 8]. Despite that, humans are the sole reservoir for *Shigella* spp., but sometimes, natural infections can also occur in captive nonhuman primates (such as macaques) [7]. Shigellosis affects individuals of all ages worldwide; however, children younger than five years bear the greatest disease burden [10]. *Shigella* spp. are transmitted predominantly through the fecal-oral route [10]. Moreover, *Shigella* spp. have been involved in several foodborne and waterborne outbreaks [4, 11]. Because of its low infectious dose (10-100 bacteria are sufficient to produce disease), *Shigella* has been a significant global health threat for many years, especially in Third World countries [4, 10]. About 5% to 10% of the diarrheal and dysentery cases are caused by different strains of *Shigella* worldwide [1, 12, 13], with the highest incidence among children ages 1 to 4 years [14, 15]. Like other disease-causing bacteria, *Shigella* spp. are the most common and first-line contagious pathogens in both affluent and developing countries in recent years [2, 11]. This infectious pathogen thrives in crowded environments, such as prisons, slums, congested colonies, and daycare centers [10, 16], and has become one of the biggest threats to public health worldwide [2, 11].

Over the course of the outbreak, diverse antibiotic resistance patterns emerged in various *Shigella* strains, complicating treatment, including recent outbreaks of multidrug-resistant (MDR) and extensively drug-resistant (XDR) strains [7, 17]. The escalating prevalence of drug-resistant *Shigella* underscores an increasingly urgent public health challenge, necessitating a unified and coordinated response. Several recent reports have affirmed that pathogenic bacteria, such as *Shigella* spp., *Klebsiella* spp., and *E. coli*, stand out as the most crucial pathogens contributing to fatalities linked to AMR [10, 18, 19]. Contiguously, antimicrobial resistance (AMR) has become a significant global concern in recent years, which is a consequent threat to public health worldwide [13, 20, 21]. Over the past few decades, the uncontrolled use of antibiotics has resulted in the emergence of MDR in bacteria [13]. MDR in *Shigella* spp. has recently been considered a significant concern in food safety and public health. The mainstay of shigellosis treatment involves antibiotic therapy, aiming to decrease the probability of complications and death while expediting clinical recovery [22]. However, it is crucial to identify the suitable antibiotics for shigellosis treatment by thoroughly comprehending the evolving resistance patterns. This entails examining the phenotypic and genotypic profiles of AMR or antimicrobial resistance genes (ARGs) in *Shigella* spp. Notably, *Shigella* serotypes are found to carry conjugative plasmids and several ARGs such as *mdt*G, *mdt*E, *amp*H, beta-lactamase, *emr*R, *emr*K, *acr*B, *tet*(D), and *tet*(A) that encode resistance to several antibiotics routinely used by the healthcare professionals [23, 24]. Moreover, *Shigella* spp. may exhibit resistance to various antibiotics through multiple mechanisms, including efflux of antibiotics, inactivation of antibiotics, diminished permeability to antibiotics, and alteration of antibiotic targets [24, 25]. The presence of such antibiotic resistance genes in *Shigella* strains is a significant public health concern, as it limits the effectiveness of commonly used antibiotics. This can complicate the treatment of *Shigella* infections and potentially lead to more severe or prolonged illness. Thus, it is essential for healthcare professionals and researchers to monitor the prevalence of antibiotic resistance in bacterial pathogens like *Shigella* and develop strategies to mitigate the spread of resistant strains. This includes prudent use of antibiotics, improved sanitation and hygiene practices, and the development of new antimicrobial agents. A series of studies through system biology approaches have been conducted to identify the ARGs by annotating bacterial whole genome sequences (WGS) and further constructed gene-drug interaction networks, protein-protein interaction (PPI) networks, and pathway enrichment analysis plots. For instance, *E. coli* O157:H7 [26], *Proteus mirabilis* [27], *K. oxytoca* [19], and *Pseudomonas aeruginosa* PA01 [28] genomes were explored through system biology and *in silico* approaches for MDR genes. However, there is no comprehensive study on gene-drug interactions, PPI, and pathway enrichment analysis among the globally circulating *Shigella* serotypes. Therefore, this study investigated 45 WGS of four *Shigella* spp. to detect ARGs and their possible mechanisms, gene-drug interactions, PPIs, and associated functional pathways. We built a phylogenetic tree to better comprehend the evolutionary divergence of the *Shigella* strains. We anticipate that the findings of this study will be useful in future clinical and pharmacological studies to design and formulate novel antimicrobials against *Shigella* like MDR and XDR pathogens.

## 2. Materials and Methods

### 2.1. Sequence Data Retrieval and Screening

Forty-five (*N* = 45) complete whole genome sequences (WGS) belonged to four species of *Shigella* such as *S. flexneri* (*n* = 17), *S. dysenteriae* (*n* = 14), *S. boydii* (*n* = 11), and *S. sonnei* (*n* = 3) were retrieved from the National Center for Biotechnology Information (NCBI) (https://www.ncbi.nlm.nih.gov/genome) (Table S1). The WGS data were obtained from the NBCI database as of June 30, 2023. We selected the genomes focusing on four *Shigella* spp. reported to cause shigellosis, a significant cause of diarrheal disease worldwide. The selection criteria are also based on their epidemiology, prevalence, antimicrobial resistance, clinical relevance, and impact on global health. Moreover, all of the genomes were sequenced from the human stool samples of the hospitalized patients from seven countries (e.g., USA, China, Tanzania, Australia, South Korea, Sweden, and Germany) of the world (Table S1). We extracted only those WGS with a read coverage depth of ≥40x. We used only assembled whole genome sequences excluding draft genomes, scaffolds, and contigs (Table S1).

### 2.2. In Silico Prediction of Antimicrobial Resistance Genes and Their Classification in Shigella spp.

The FASTA files for the individual genome were screened to detect ARGs, classes of resistant antibiotics, and concurrent resistance mechanisms. The ABRicate v1.0.1 (https://github.com/tseemann/abricate) bundled with multiple databases, NCBI AMRFinderPlus [29], CARD 2020 [30], ARG-ANNOT [31], ResFinder 4.0 [32], and MEGARes 2.0 [33], was used to predict ARGs in the assembled genomes. The ARG selection criteria were set to perfect (100% identity) and strict (>95% identity) hits only to the curated reference sequences in the databases. We further utilized an open-accessed software, Venny 2.1 (https://bioinfogp.cnb.csic.es/tools/venny/, accessed on June 30, 2023), for mapping and comparing ARG lists and to identify the unique gene list within a long list of ARGs that may be used interactively. The abundance of ARGs was calculated by using the RGI program to identify drug-resistant genes by comparison with the reference genome in the CARD (https://card.mcmaster.ca/, accessed on June 30, 2023). Finally, we used these unique ARGs to build gene interaction networks and further analysis.

### 2.3. Phylogenetic Relationship of Shigella spp.

To elucidate the evolutionary relationship among the studied *Shigella* genomes (*n* = 45), a maximum-likelihood (ML) phylogenetic tree was constructed. MEGA (Molecular Evolutionary Genetics Analysis) v7.0 [34] and NCBI Tree Viewer (https://www.ncbi.nlm.nih.gov/tools/treeviewer/, accessed on July 10, 2023) tools were used to construct the phylogenetic tree applying ML method with 1000 bootstraps. The phylogenetic tree was subsequently imported to interactive tree of life (iTOL) v. 3.5.4 (http://itol.embl.de/, accessed on July 10, 2023) for better visualization, and bootstra*p* values were reported for each branch [35]. Each node in the phylogenetic tree represents an isolate, while the edge represents the Hamming distance between two isolates.

### 2.4. Functional Domains, Protein Featuring Pathway, and Protein-Protein Interaction Analysis

GO (Gene Ontology) database [36] and KEGG (the Kyoto Encyclopedia of Genes and Genomes) [37] databases were used to predict the functions of the detected ARGs. We utilized Enrichr [38] with Fisher's exact test to conduct the functional enrichment analysis of the study genomes. We retrieved GO keywords and KEGG pathway's data from the STRING v11.5 (https://string-db.org/) database (accessed on July 12, 2023) and then utilized SRplot (http://www.bioinformatics.com.cn/en) to visualize the results. We used Pfam [39] and InterPro [40], for protein domain analysis. The Science and Research online plot (SRplot) (http://www.bioinformatics.com.cn/srplot, accessed on July 12, 2023) tool was used for generating different types of plots to analyze statistically figure generation and finding correlations among different entities. To assess the relationships between ARGs in the genomes of *Shigella*, we constructed a PPI network by using the Search Tool for the Retrieval of Interacting Genes (STRING v11.0) [41]. To eliminate inconsistent PPIs from the dataset, we set a cut-off criterion to a high confident interaction score of ≥0.7. Thereafter, we incorporated the results from the STRING database into Cytoscape v3.9.1 (https://cytoscape.org/, accessed on July 13, 2023) to create a visual representation of the target network for molecular interactions [42] and to envisage the PPIs within the statistically significant ARGs. A *p* value < 0.01 was considered statistically significant.

### 2.5. Cluster Analysis and Selection of Hub Proteins

We utilized the molecular complex detection (MCODE) plugin from Cytoscape to identify the interconnected regions or clusters from the PPI network [43]. The cluster finding parameters were adopted, such as a degree cut-off of 2, a node score cut-off of 0.2, a kappa score (*K*-core) of 2, and a maximum depth of 100 in the MCODE, which limits the cluster size for coexpressing networks and verifies the efficacy of interactive collaborators in the context of ARG expression [43]. We further utilized the Cytoscape plug-in CytoHubba to find highly interconnected protein nodes and investigate the network topology. Our analysis included five approaches using the CytoHubba plugin, including three locally ranked methodologies, degree, MNC (maximum neighbourhood component), and MCC (maximum clique centrality), as well as two globally ranked methodologies, closeness and betweenness [44]. Subsequently, collected ARGs from the CytoHubba were submitted to jvenn (interactive Venn diagram analyzer) for further analysis. The ARGs that were intersected among the six approaches of CytoHubba were considered as significant hub proteins [44].

### 2.6. Network Construction for Antimicrobial Resistance Genes, Antibiotic Classes, and Resistance Mechanisms

To briefly visualize the ARG resistance mechanism, ARG antibiotic class, and antibiotic class resistance mechanism interaction networks, we constructed a sunburst plot and bar diagrams using Python. We used high-level programming languages to create extraordinary Python libraries, including Pandas, NumPy, SciPy, Matplotlib, and Plotly, for data analysis and visualization [45].

### 2.7. Statistical Analyses

The majority of statistics for this project are descriptive in nature. For comparison of the distribution of ARGs, resistance mechanisms, gene-drug interactions, and PPI traits across the genomes of different *Shigella* spp., Student's *t*-tests were used with statistical significance observed at *p* < 0.05.

## 3. Results

### 3.1. Genome Sequences, Phylogenetic Relationship, and Resistance Repertoire of Shigella spp.

**Figure 1:**
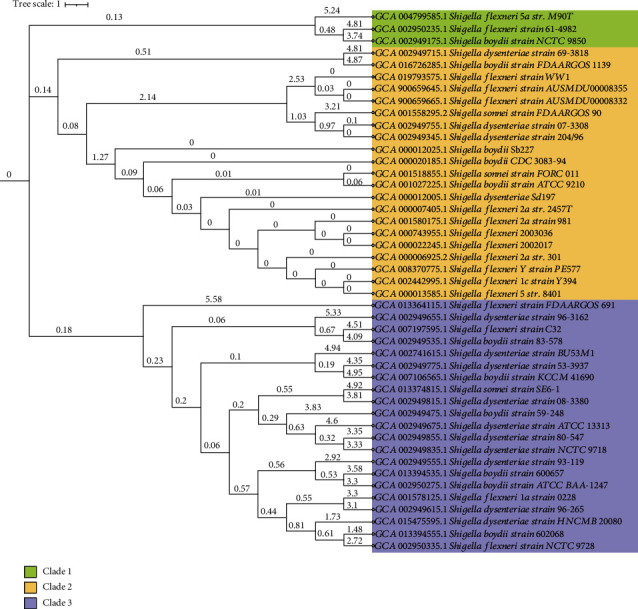
The evolutionary phylogenetic relationships among different strains of *Shigella*. Genomes of 45 *Shigella* strains (*S. flexneri*, *n* = 17; *S. dysenteriae*, *n* = 14; *S. boydii*, *n* = 11; and *S. sonnei*, *n* = 13) were used to construct the phylogenetic tree applying maximum-likelihood method with 1000 bootstraps. The tree topology partitions the isolates into three distinct clades (clade 1–clade 3). Each node in the phylogenetic tree represents an isolate while the edge represents the hamming distance between two isolates.

We found 25,002 complete genomes of *Shigella* spp. in the NCBI GenBank database up to June 30, 2023. Through comprehensive filtering in terms of genome coverage, we selected only 45 complete genomes with a genome coverage of ≥40x, and size of the selected genomes ranged from 4.5 to 5.2 mega base pairs (Table S1). The selected genomes belonged to four species of *Shigella*, of which 37.78% (17/45), 31.11% (14/45), 24.44% (11/45), and 6.67% (3/45) genomes were from *S. flexneri*, *S. dysenteriae*, *S. boydii*, and *S. sonnei*, respectively. To determine the evolutionary relationship across the genomes of *S. flexneri*, *S. dysenteriae*, *S. boydii*, and *S. sonnei*, we established a maximum-likelihood phylogenetic tree (Figure 1). The phylogenetic tree showed three main clades among 45 genomes, with each supported by 100% bootstrapping (Figure 1). In this phylogenetic tree, clade 1 consisted of only three strains (two *S. flexneri* and one *S. boydii*) of *Shigella*, whereas clade 2 and clade 3 possessed 21 strains of *Shigella* (in each). We also observed that every ramification of nodes describes each strain of *Shigella* (Figure 1), implying no significant heterogeneity among the circulating strains of four *Shigella* spp. Furthermore, the genetic homogeneity of these clusters was evidenced by the high degree of overall nucleotide sequence similarity in the genomes. Through a comprehensive annotation of these genomes, we detected 2,146 ARGs including 835 ARGs in *S. flexneri* strains, 620 ARGs in *S. dysenteriae* strains, 519 ARGs in *S. boydii* strains, and 172 ARGs in *S. sonnei* strains. Out of 2,146 detected ARGs, 91 genes were found to be shared among the genomes of four *Shigella* spp. Therefore, these 91 shared ARGs were implemented to explore this current study further. Among the selected genomes, ARGs counts ranged from 38 to 61 with an average count of 47.69 ARGs per genome (Figure S1). In this study, we identified a higher proportion of ARGs coding for fluoroquinolones, penems, cephalosporins, tetracyclines, aminoglycosides, and macrolide resistance in related *Shigella* spp. (Figure S2). By calculating the number of ARGs against particular antibiotics, we found that 30 genes showed resistance against fluoroquinolone antibiotics followed by 27 genes against cephalosporins and penems (Figure S2). Besides, 21, 16, 15, 13, and 11 ARGs showed resistance to tetracyclines, aminoglycosides, phenolics, and rifamycin antibiotics, respectively (Figure S2).

### 3.2. Protein-Protein Interaction and Clustering Show Potential Hub Proteins in Shigella Genomes

**Figure 2:**
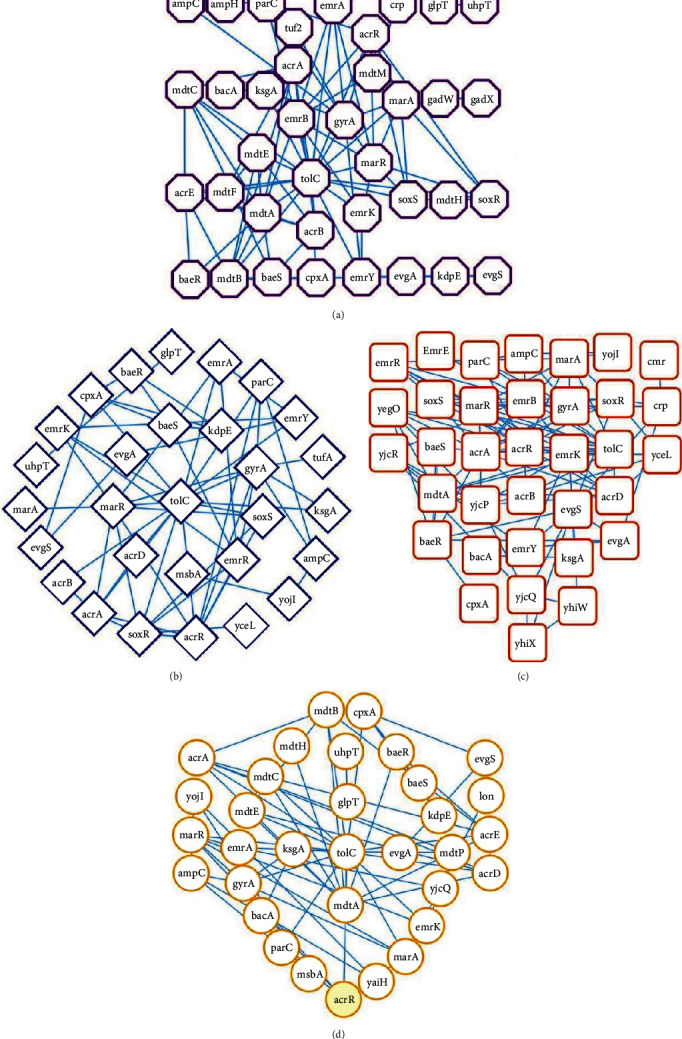
Protein-protein interaction (PPI) network connectivity and gene interaction network of the most significant shared ARGs with their interacting partners. The network contains (a) 38 nodes with 90 edges for *S. boydii*, (b) 29 nodes with 57 edges for *S. dysenteriae*, (c) 35 nodes with 108 edges for *S. flexneri*, and (d) 33 nodes with 67 edges for *S. sonnei*. Edges represent the protein-protein associations. Node size indicates node degree value, and edge colour and shape represent *Shigella* spp.

**Figure 3:**
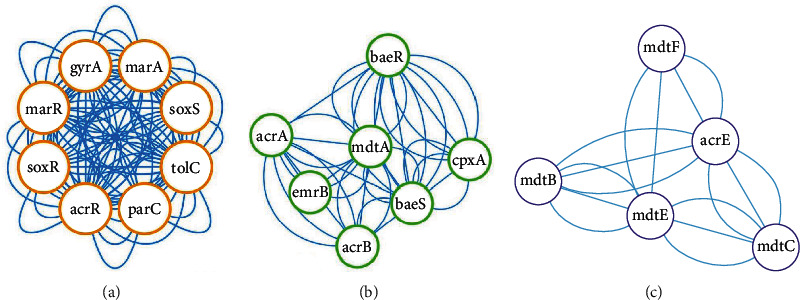
Clustering of protein-protein interaction (PPI) networks. The PPI networks were merged into three clusters: (a) the 1^st^ cluster comprised 8 nodes with 94 edges, (b) the 2^nd^ cluster comprised 7 nodes with 39 edges, and (c) the 3^rd^ cluster consisted of 5 nodes with 16 edges. These clusters had the highest scores among the clusters.

To predict typical PPI network connectivity, protein interactions and hub proteins were explored through the STRING database and visualized using Cytoscape (Figure 2). In a PPI network, a node typically represents a protein name, identifier, and other attributes depending on the context of the network analysis. On the other hand, edge represents the biochemical or physical interaction between the two proteins and also the strength of interaction. The PPI network possessed 38 nodes and 90 edges for *S. boydii* (Figure 2(a)), 29 nodes and 57 edges for *S. dysenteriae* (Figure 2(b)), 35 nodes and 108 edges for *S. flexneri* (Figure 2(c)), and 33 nodes and 67 edges for *S. sonnei* (Figure 2(d)). We detected three significant clusters by merging four PPI networks of *Shigella* spp. through the MCODE plugin of Cytoscape, based on scoring function (Table 1). The score in MCODE represents the “cliquishness” or the tightness of connections within a cluster. Higher scores generally indicate denser and more significant clusters, meaning that the nodes within those clusters are more tightly interconnected compared to nodes outside the cluster. Within these clusters, cluster 1 comprised 8 nodes and 94 edges (Figure 3(a) and Table 1) and cluster 2 comprised 7 nodes and 39 edges (Figure 3(b) and Table 1), whereas cluster 3 consisted of 5 nodes and 16 edges (Figure 3(c) and Table 1).

**Figure 4:**
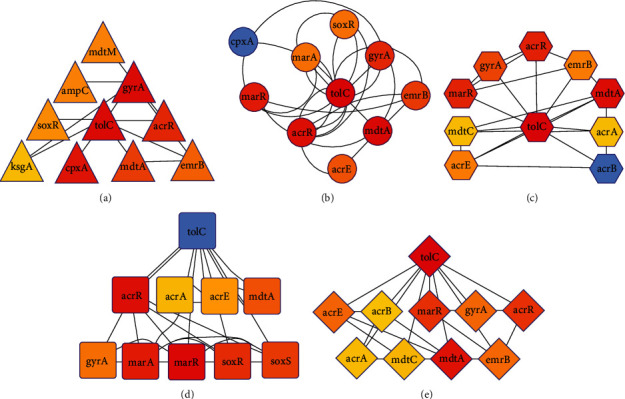
Visualization of the hub genes from the protein-protein interaction (PPI) network using five different calculation methods: (a) betweenness, (b) closeness, (c) degree, (d) maximal clique centrality (MCC), and (e) maximum neighbourhood component (MNC).

**Figure 5:**
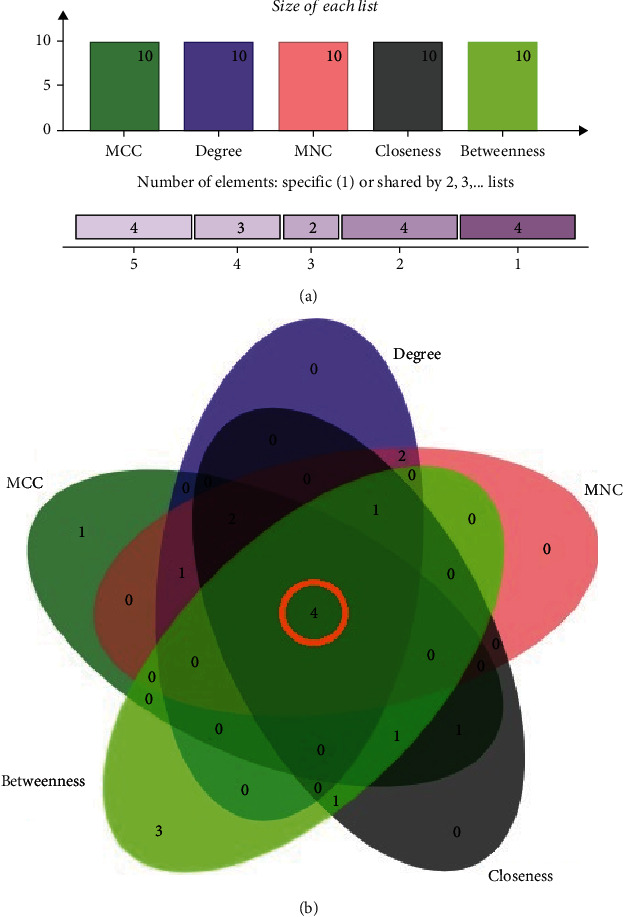
Visualization of the hub genes using five different calculation methods. (a) Bar plot to identify significant top 13 hub genes using five intersecting algorithms (betweenness, closeness, degree, MCC, and MNC) for differentially expressed ARGs in *Shigella* genomes. (b) Four hub proteins (e.g., tolC, acrR, mdtA, and gyrA) were found to be shared in the five methods.

In this study, we detected hub proteins by employing five different methodologies such as betweenness (Figure 4(a)), closeness (Figure 4(b)), degree (Figure 4(c)), MCC (Figure 4(d)), and MNC (Figure 4(e)). A total of 13 proteins such cpxA, soxR, emrB, gyrA, marR, marA, acrR, acrE, tolC, mdtA, mdtC, acrB, and acrA were identified as hub proteins (ten proteins in each method) (Figure 5(a) and Table 2). Among these proteins, only four hub proteins (tolC, acrR, mdtA, and gyrA) were common in the five methods (Figure 5(b)). Importantly, marR and acrE proteins were detected in four methods except for betweenness (Figure 4(b)), while emrB was found in four methods except for the MCC algorithm (Figure 4(d)). The topological properties of unique hub proteins are shown in Table 2. Moreover, the identified hub proteins were also detected in cluster 1, cluster 2, and cluster 3 (as mentioned in Figure 3), indicating that they were the most crucial hub proteins. Our PPI networking and clustering analysis revealed that these hub proteins might have the potential to function in antibiotic-resistant mechanisms and/or pathways.

### 3.3. Gene Ontology, KEGG Pathway, and Protein Domain Analyses Reveal Significant Pathway and Domain Features

**Figure 6:**
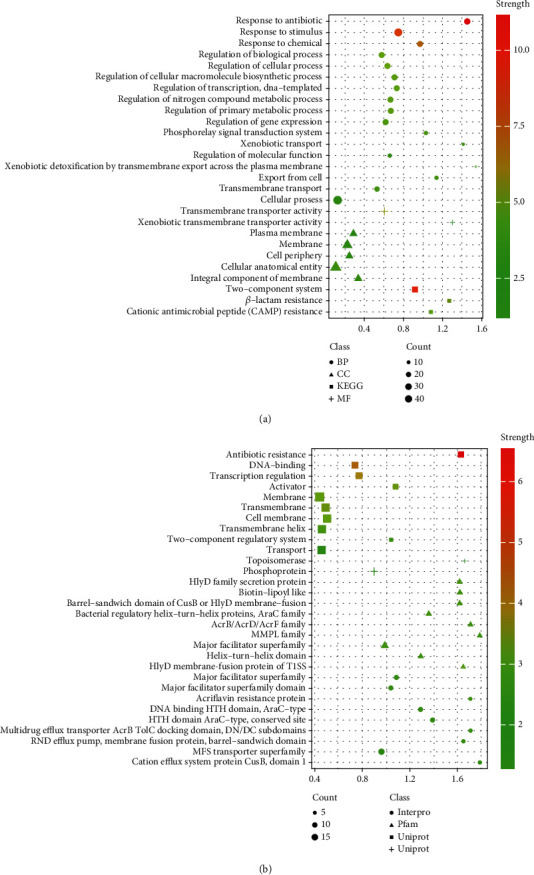
Gene Ontology (GO) and KEGG pathway enrichment analyses of differentially expressed ARGs. (a) The top 27 most enriched GO terms found in the analysis of ARGs. (b) KEGG pathway enrichment analysis of ARGs showing the top 30 functional pathways.

To analyze the functions of the detected ARGs and further understand their resistance mechanism regulation, we performed GO assignment and enrichment and KEGG functional analyses. The detected ARGs were assigned to and enriched in one or more of three categories: biological process (BP), molecular function (MF), and cellular component (CC) with 3 to 45 gene counts (Figure 6(a)). The ARGs were significantly enriched (*p* < 0.05 and strength 0.11 to 1.54) in 24 GO terms. Of these enriched GO terms, 17 were related to BP whereas five to CC and two to MF, respectively (Figure 6(a)). The most significant and enriched GO terms of the BP were cellular process, response to stimulus, regulation of biological process, regulation of cellular process, and response to chemicals and antibiotics, and those of MF were transmembrane transporter activity and xenobiotic transmembrane transporter activity (Figure 6(a)). Besides, the top four significantly enriched GO terms of the CC were cellular anatomical entities, membrane, cell periphery, and cellular component : plasma membrane, suggesting that AMR genes are regulated by multiple cellular processes (Figure 6(a)). The two-component system, cationic antimicrobial peptide (CAMP) resistance, and beta-lactam resistance were the most enriched KEGG pathways related to the citrate cycle (TCA cycle), the breakdown of RNA, the metabolism of carbon, and the production of secondary metabolites (Figure 6(a)). These results and observations suggested that *Shigella* spp. were involved in regulating various metabolic pathways to develop antimicrobial resistance. In order to understand the importance of domain associations in *Shigella* spp., high-frequently co-occurrent domains and their interactions were systematically identified, which were then interpreted for their contributions to bacterial-specific antimicrobial resistance (AMR) features. We calculated and filtered out the distinct pathways to generate a bubble plot to present the significant correlation. In this study, gene count ranged from 2 to 18, strength values varied from 0.44 to 1.79, and *p* values ranged from 0.0074 to 3.97*E*-07 (Figure 6(b)). Highly AMR-associated Pfam domains, corresponding antibiotic resistance, transcription regulation, membrane, transmembrane and cell membrane activities, two-component regulatory systems, major facilitator superfamily, and MFS transporter superfamily were the significant protein domains identified in the *Shigella* spp. genome (Figure 6(b)).

### 3.4. Antibiotic Resistance Genes, Drug Class, and Resistance Mechanisms Are Correlated in Shigella spp.

By analyzing the ARG repertoire of the individual genome, we found that each of the *Shigella* genome encoded multiple ARGs that confer various types of resistance mechanisms and several strategies to raise their resistance capabilities against multiple drugs. By comparing the mechanisms of resistance conferred by the detected ARGs (*n* = 2,146) in *Shigella* genomes, we found that 51.0% of ARGs conferred their resistance through antibiotic efflux mechanisms, whereas 23, 18, 5, 2, and 1% ARGs mediated their resistance through antibiotic target alteration, antibiotic target replacement, reduced permeability to antibiotics, and antibiotic target protection, respectively (Figure S3). Further investigation into the shared ARGs (*n* = 91) revealed that these ARGs conferred resistance against 24 distinct antibiotics belonging to 16 drug classes. We found a higher number of shared ARGs conferring resistance through efflux pump mechanism (*n* = 46) followed by antibiotic inactivation (*n* = 21), antibiotic target alteration (*n* = 16), antibiotic target replacement (*n* = 5), reduced permeability to antibiotics (*n* = 2), and antibiotic target protection (*n* = 1) mechanisms (Figure S4). In this study, we figured out four hub genes, e.g., *tol*C, *acr*R, *mdt*A, and *gyr*A, from 91 shared ARGs, and of these hub genes, *tol*C and *mdt*A operate antibiotic efflux mechanisms. In contrast, *acr*R and *gyr*A genes wield antibiotic target alteration mechanisms to inhibit antibiotic sensitivity (Figure S4).

## 4. Discussion

*Shigella* is the leading global etiological agent of shigellosis, a potential public health catastrophe globally. The prevalence of MDR and/or XDR *Shigella* spp. is increasing and becoming globally dominant in shigellosis [2, 10, 46]. Moreover, *Shigella* spp. are on the priority list of the World Health Organization regarding antimicrobial resistance [47]. Therefore, shigellosis is becoming critical day by day and necessitate new interventions for prevention, treatment, and control. The current global landscape of shigellosis is dominated by *S. flexneri* and *S. sonnei*, with *S. sonnei* and *S. boydii* clearly in the ascendency in many low- and middle-income countries [2, 10, 46]. The advancement in WGS and the application of online bioinformatics tools for real-time detection of AMR determinants, ARGs, and mechanism of resistance are essential to identify effective control and prevention strategies to combat the increasing threat of AMR especially in bacterial diseases caused by MDR or XDR lineages [47, 48]. However, until now, no studies have explored ARGs and their mechanism of resistance, gene-drug interactions, PPI, and functional pathways in *Shigella* spp. By utilizing 45 WGS of four species of *Shigella* spp. (e.g., *S. flexneri*, *S. dysenteriae*, *S. boydii*, and *S. sonnei*), we identified four hub proteins (e.g., tolC, acrR, mdtA, and gyrA) that could be exploited to design and develop novel therapeutic candidate(s) against MDR or XDR lineages of *Shigella* spp.

The phylogenetic analysis with 45 genomes showed three distinct clades among the circulating strains of *Shigella* worldwide. However, no significant heterogeneity was observed among the circulating strains of *Shigella*. The genetic homogeneity of these clusters was evidenced by the high degree of overall nucleotide sequence similarity in the study genomes. Our results are consistent with a previous study based on inferred bacterial coconserved networks based on phylogenetic profiles [49]. By conducting an extensive search and utilizing bioinformatics analysis, we identified 2,146 ARGs across 45 genomes, averaging 47.69 ARGs per genome. Among these ARGs, only 91 ARGs were found to be shared by the four species of *Shigella*. We found that higher proportion of ARGs was related to fluoroquinolones, penems, cephalosporins, tetracyclines, aminoglycosides, and macrolide resistance. Antimicrobial resistance to *Shigella* spp. is a growing international concern, specifically with the international dominance of the MDR or XDR lineages [2, 48]. AMR determination by WGS approaches can complement traditional laboratory-based surveillance and provide direct insights into their evolution and transmission from one strain to another [50, 51]. The high throughput WGS data can help reveal the ARGs and their possible mechanism for drugs not being tested routinely or where the mechanisms of antimicrobial resistance are not yet identified [52, 53].

We also sought to predict PPI network connectivity, ARG interactions, and hub genes which revealed significant clustering among the strains of four *Shigella* spp., with highly connected networks and node interactions. The PPI networks seem to be the most well-connected one with around 30 nodes, at least 60 connections per species, and relatively high clustering coefficient. Understanding the PPI networks among *Shigella* genomes and the strength of association between two or more proteins makes it easier to select drug targets. The high clustering coefficient indicates highly connected networks, while node degree denotes the number of interactions the network proteins have on average [54]. The merged network, subjected to cluster analysis, showed that the proteins fall into three clusters with significant variations. Therefore, we suggest that the PPI networks that show a higher number of interactions could be more critical in analyzing the molecular-level interactions of ARGs as also reported earlier [28]. The PPI network analysis performed using these ARGs showed that there were several unique genes conferring AMR in different strains of *Shigella* through different resistance mechanisms. We also detected four hub genes such as *tol*C, *acr*R, *mdtA*, and *gyr*A showing significant interactions. These genes play numerous essential roles in the metabolic, cellular, and biological processes that occur in microbes [55]. Previously, some researchers described that *tolC*, *acrR*, *mdtA*, and *gyrA* genes in *Shigella* strains plied different resistant mechanisms to survive [2, 4, 7]. The overexpression of efflux pump-related genes (e.g., *acrR*, *TolC*, and *mdtA*) may cause an overall decreased accumulation of antibiotics inside the bacterial cells, further resulting in decreased susceptibility and development of MDR or XDR phenomena [2, 56]. Moreover, *mdtA* is a novel immunogenic *Shigella* protein responsible for the efflux pump-mediated AMR in *Shigella* [22, 57]. In addition, *acrR* and *gyrA* gene expression implied an antibiotic target alteration and reduced antibiotic sensitivity [58]. The mutations in bacterial targets *gyrA* and *gyrB* encoding for DNA gyrase and topoisomerase IV cause a change either in the target structure or its binding strength, resulting in less susceptibility, and increased minimum inhibitory concentrations [4]. We found that there was a strong link between these hub genes and different types of drug resistance mechanisms and drug classes. These mechanisms can be carried out by changing the drug, changing the antimicrobial targets, limiting access to the target, or a combination of these things [4, 13]. Moreover, predicted ARGs can easily spread through their host bacteria to different host inhabitants of other ecosystems [53, 59].

One of the hallmark findings of this study is the prediction of both ARG repertoire and correlated biological functions (e.g., biological processes (BP), cellular components (CC), and molecular functions (MF)) through which different strains of *Shigella* develop AMR. Gene Ontology enrichment analysis of the ARGs in *Shigella* genomes showed that majority of the shared ARGs represented biological functions involved mainly in BP, MF, and CC. The cellular metabolic process of bacteria determines their resistance to antibiotics; hence, the metabolic condition of bacteria could be modified to boost therapeutic efficacy [60]. Disruptions to the bacterial metabolic balance also significantly affect treatment plans [60, 61]. We found that two-component system, primary metabolic process, and nitrogenous compound metabolism were the most enriched pathways in different strains of *Shigella*. Our results therefore suggest that *Shigella* spp. were involved in regulating various metabolic pathways, which might play a significant role in the development of resistance to multiple antibiotics. For instance, the two-component regulatory system plays a substantial role in the pathogenicity, virulence, biofilm formation, and drug resistance in bacterial pathogens, including *Shigella* [62].

Another important finding of the current study is that we found significant correlation across the detected ARGs, drug class, and resistance mechanisms. We identified that *Shigella* spp. developed AMR through efflux pump mechanism, antibiotic inactivation, antibiotic target alteration, antibiotic target replacement, reduced permeability to antibiotics, and antibiotic target protection mechanisms. Various antibiotic resistance mechanisms in bacterial pathogens include poor drug penetration into the cell, efflux of antibiotics by efflux pumps, target modification by mutation, and hydrolysis of antibiotics [13, 63]. It has been reported that efflux pumps regulated by two-component systems in several bacterial pathogens provide multidrug resistance, which may limit the treatment options against bacterial infections like shigellosis. Furthermore, antibiotic accumulation is known to be one prominent feature of bacterium tolerance. Our findings showed that efflux pump, antibiotic inactivation, antibiotic target alteration, replacement, protection, and reduced permeability to antibiotics were the major activities playing significant roles in AMR development in *Shigella* spp. Our findings also suggest that specific pumps are involved in tolerance maintenance, which is a new concept in antibiotic tolerance studies. Therefore, a crucial aspect is the thorough characterization of sources, reservoirs, mechanisms, and networks involved in the potential transmission of AMR and ARGs among humans, animals, and the environment [64]. This comprehensive understanding is pivotal for devising interventions that are both efficient and effective in addressing pathogens resistant to antimicrobials [13].

## 5. Conclusions

Antimicrobial resistance in human pathogenic bacteria such as *Shigella* is a complex and multifaceted challenge. We unveiled noteworthy genomic characteristics in MDR *Shigella* spp., including *S. flexneri*, *S. dysenteriae*, *S. boydii*, and *S. sonnei*. These findings have implications for devising novel therapeutic approaches aimed at preventing, treating, and controlling shigellosis. Evolutionary phylogenetic analysis revealed three distinct clades in 45 strains of *Shigella*, with less genomic diversity. We found a significant correlation among ARGs, their resistance mechanisms, and drug classes in the genomes of four *Shigella* species. Several GO keywords and KEGG pathways correlated with MDR were identified during the functional enrichment analysis of the ARGs. In *Shigella* spp., ARGs and their functional interactions most commonly expressed included antibiotic resistance through the inactivation of antibiotics, antibiotic efflux pump, target alteration, reduced permeability to antibiotics, and target replacement of antibiotics. Numerous antibiotics such as cephalosporins, penems, fluoroquinolone antibiotic, tetracyclines, aminoglycoside antibiotics, monobactam, carbapenems, macrolides, and sulfonamides are found to be ineffective to *Shigella* spp. Besides, PPI clustering revealed that some of the ARG sets are closely connected to develop resistance in *Shigella* spp. We identified four unique hub genes (e.g., *tol*C, *acr*R, *mdt*A, and *gyr*A) among the ARG repertoire of *Shigella*, and these hub genes could be used as potential therapeutic candidates and aid in developing new drugs. Therefore, *Shigella* spp. harbored a higher number of ARGs encoding for different resistance mechanisms, which must be considered for further research. Our findings will thereby furnish researchers with a firm foundation to develop hypothesis to predict clinically significant determinants of antibiotic resistance. This will, in turn, support investigations into innovative therapeutic strategies for effectively managing shigellosis outbreaks on a global scale.

## Figures and Tables

**Table 1:** Protein-protein clustering information of *Shigella* genomes.

Cluster no.	Node number	Edge number	Score	Proteins
Cluster 1	8	94	6.286	gyrA, marA, soxS, tolC, parC, acrR, soxR, marR
Cluster 2	7	39	3.333	baeR, acrA, emrB, acrB, baeS, cpxA, mdtA
Cluster 3	5	16	3	mdtB, mdtE, mdtC, acrE, mdtF

Here, clustering scores are solely based on nodes/proteins.

**Table 2:** Topological information of hub proteins as determined by five distinct algorithms.

Proteins	Full name	MCC	MNC	Degree	Closeness	Betweenness	Stress
cpxA	Sensor histidine kinase	6	3	5	17.67	124.93	230
soxR	Superoxide response regulon	121	5	6	17.75	64	118
emrB	Multidrug resistance protein B	22	7	7	18.58	67.167	162
gyrA	DNA gyrase subunit A	35	7	8	19.33	145.73	322
marR	Multiple antibiotic resistance regulator	162	9	9	19.67	26.3	78
marA	Multiple antibiotic resistance activator	144	6	6	18.17	5	24
acrR	Acriflavine resistance regulator	158	9	9	19.83	79.93	184
acrE	Acriflavine resistance protein E	26	7	7	18.33	17.73	66
tolC	Outer membrane efflux protein	249	20	21	26.67	623.5	1,114
mdtA	Multidrug transporter protein A	84	11	11	20.58	68.67	202
mdtC	Multidrug transporter protein C	20	6	6	17.67	8.93	50
acrB	Acriflavine resistance protein B	20	6	6	17.67	8.93	50
acrA	Acriflavine resistance protein A	24	6	6	17.67	2.2	12

MCC: maximum clique centrality; MNC: maximum neighbourhood component.

## Data Availability

The WGS data of the *Shigella* spp. used in this study are available in the public database (GenBank, NCBI) under different accession numbers. The detailed information on the study genomes is available in Table S1.

## Supplementary Materials

Supplementary Materials All relevant supplementary data (Figure S1–S4 and Table S1) are included in the manuscript. Further inquiries may be directed to the corresponding author (nazmul90@bsmrau.edu.bd).
