# Supplementary material for: Genome-Wide Investigation Reveals Potential Therapeutic Targets in Shigella spp
Source: Biomed Res Int. 2024 Mar 21;2024:5554208. doi: 10.1155/2024/5554208 (PMC11003385; doi:10.1155/2024/5554208)
Supplement: Supplementary Materials — All relevant supplementary data (Figure S1–S4 and Table S1) are included in the manuscript. Further inquiries may be directed to the corresponding author (nazmul90@bsmrau.edu.bd). [file 5554208.f1.pdf]

Supplementary File

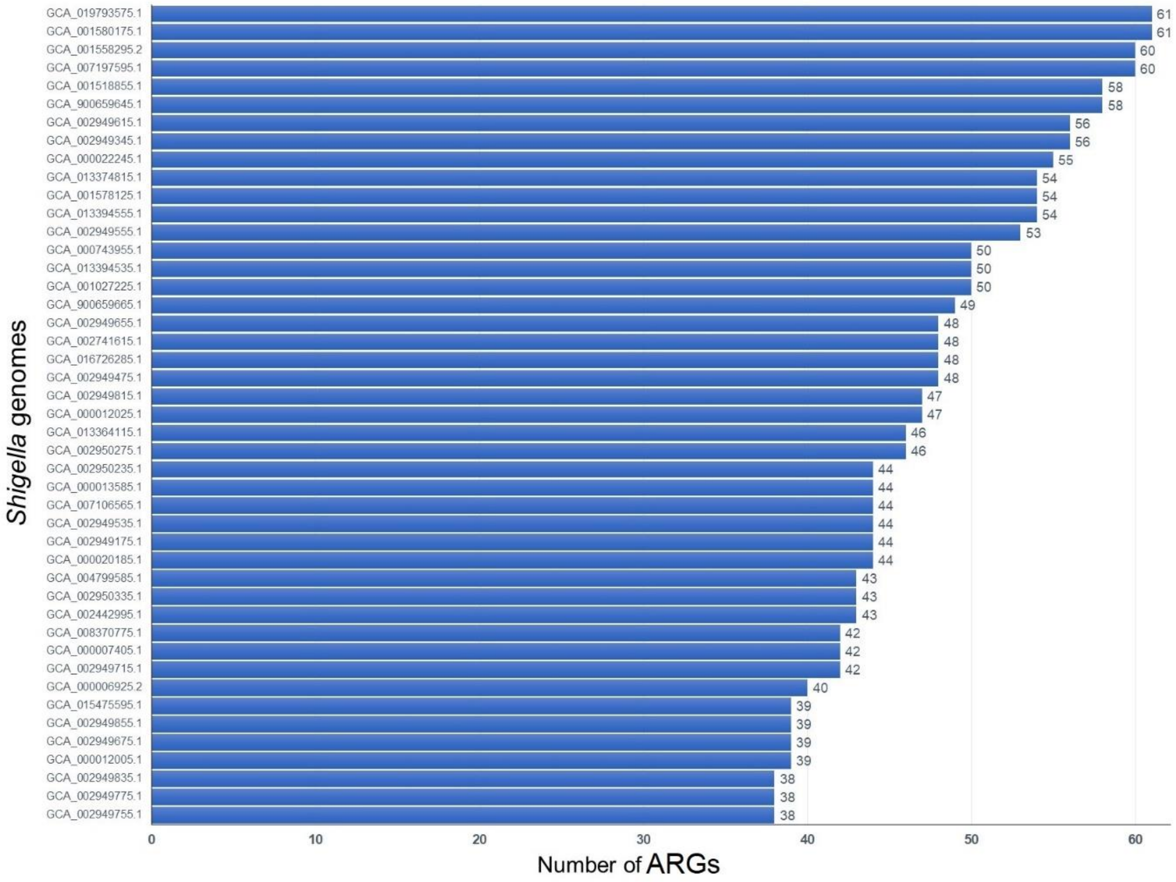

Figure S1. Number of antibiotic resistance genes (ARGs) detected in the genomes of the *Shigella* spp. strains. The X-axis represents the number of ARGs and Y-axis shows the name of the isolates of the corresponding *Shigella* genomes.

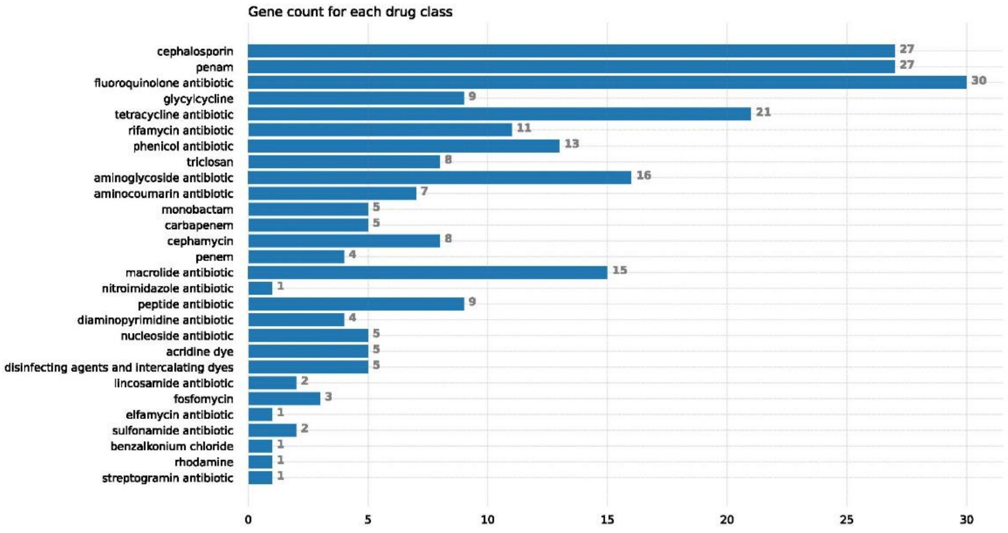

Figure S2. Antibiotic resistance gene (ARG) counts against different drug classes detected in the genomes of four *Shigella* species. The X-axis represents ARG counts and Y-axis shows the different drug classes.

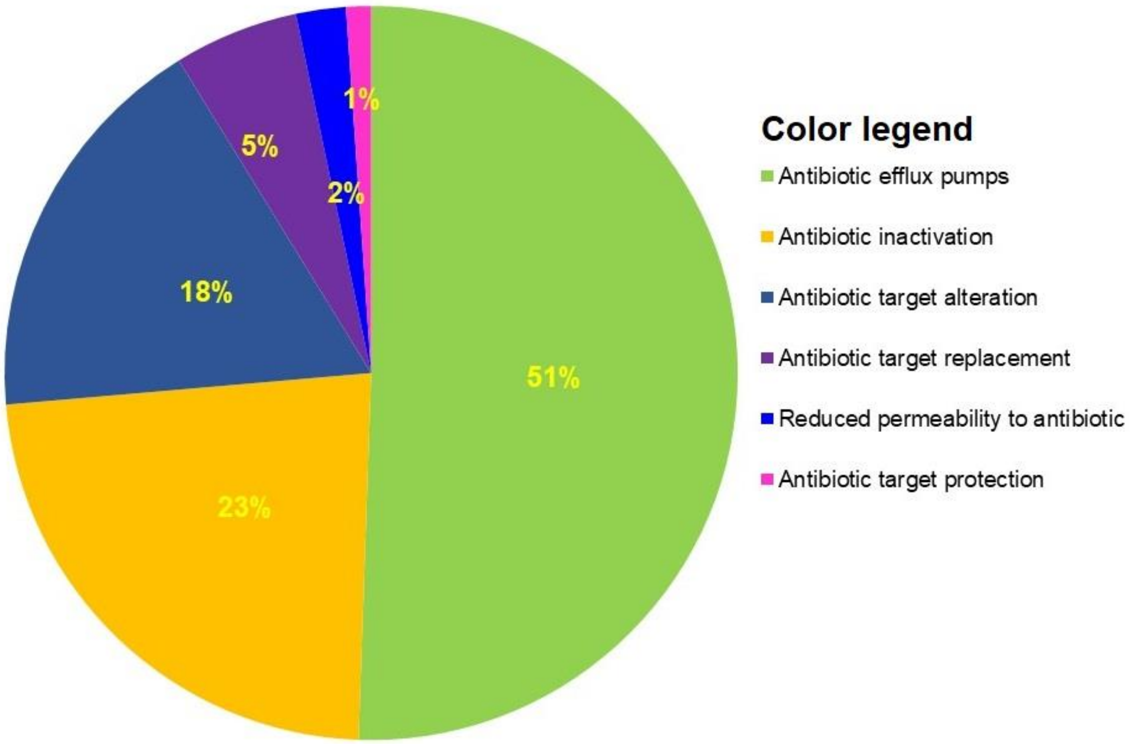

Figure S3. Different antibiotic resistance mechanisms showed by *Shigella* spp.

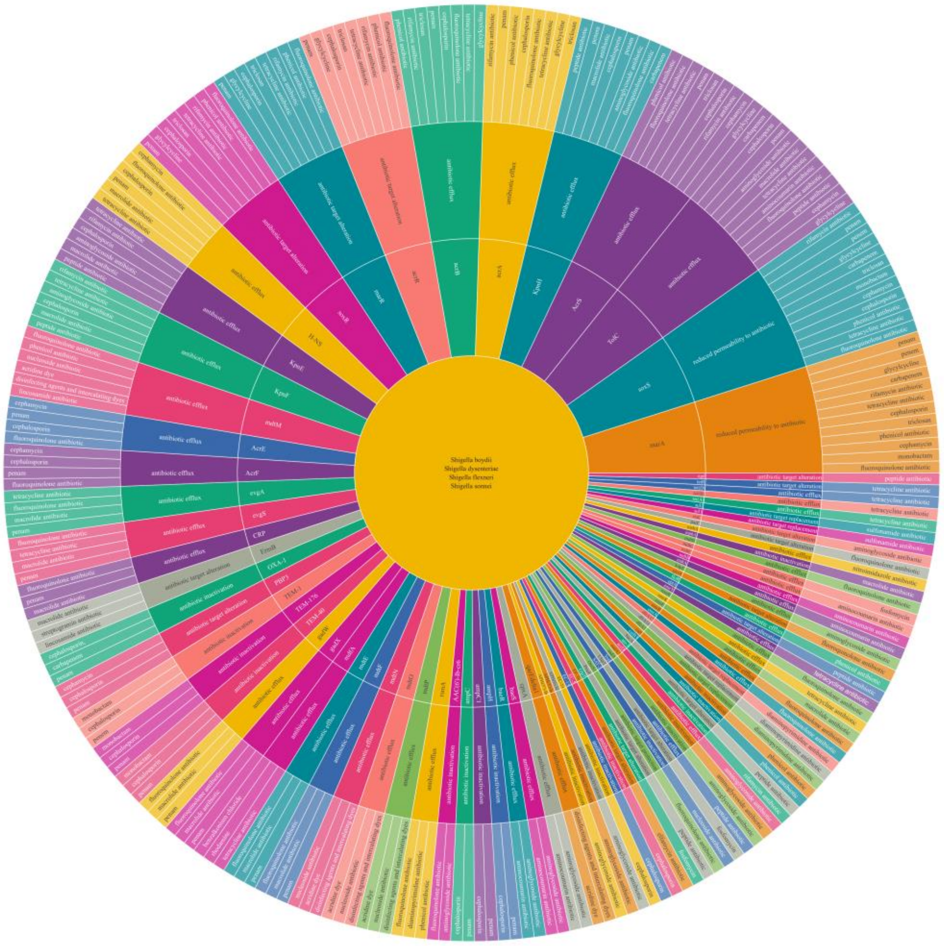

Loading [MathJax]/extensions/MathMenu.js

Figure S4. Sun burst plot showing 91 shared ARGs, antibiotics, drug classes, and their mechanisms in 45 genomes of *Shigella*.

Table S1. Study genomes information.

Sl. No	Accession No.	*Shigella* spp.	Country of origin	Genome coverage	Genome size (Mb)	Host/Sample	Source	Number of gene	Reference
1	GCA_000006925.2	*S. flexneri*	China	Not provided	4.829	Human/Stool	Hospital patient	4,788	NCBI:txid198214
2	GCA_000007405.1		USA	Not provided	4.599	Human/Stool	Hospital patient	4,618	NCBI:txid198215
3	GCA_000013585.1		China	Not provided	4.574	Human/Stool	Hospital patient	4,609	NCBI:txid373384
4	GCA_000022245.1		China	Not provided	4.894	Human/Stool	Hospital patient	4,984	NCBI:txid591020
5	GCA_000743955.1		China	Not provided	4.596	Human/Stool	Hospital patient	4,635	NCBI:txid1282357
6	GCA_001578125.1		China	164.55	4.853	Human/Stool	Hospital patient	4,978	NCBI:txid374923
7	GCA_001580175.1		China	58x	4.892	Human/Stool	Hospital patient	4,980	NCBI:txid42897
8	GCA_002442995.1		Australia	203.0x	4.817	Human/Stool	Hospital patient	4,910	NCBI:txid1935181
9	GCA_002950235.1		Tanzania	127.0x	4.691	Human/Stool	Hospital patient	4,763	NCBI:txid623
10	GCA_002950335.1		Tanzania	135.0x	4.646	Human/Stool	Hospital patient	4,710	NCBI:txid623
11	GCA_004799585.1		Sweden	155.0x	4.829	Human/Stool	Hospital patient	4,917	NCBI:txid1086030
12	GCA_007197595.1		South Korea	166.0x	5.127	Human/Stool	Hospital patient	5,015	NCBI:txid623
13	GCA_008370775.1		USA	30.0x	4.599	Human/Stool	Hospital patient	4,626	NCBI:txid424720
14	GCA_013364115.1		USA	846.51x	4.544	Human/Stool	Hospital patient	4,564	NCBI:txid623
15	GCA_019793575.1		China	400.0x	4.834	Human/Stool	Hospital patient	4,635	NCBI:txid623
16	GCA_900659665.1		Australia	225x	4.944	Human/Stool	Hospital patient	5,047	NCBI:txid623
17	GCA_900659645.1		Australia	181x	4.616	Human/Stool	Hospital patient	4,675	NCBI:txid623
18	GCA_000012005.1	*S. dysenteriae*	China	Not provided	4.561	Human/Stool	Hospital patient	4,840	NCBI:txid300267
19	GCA_002741615.1		Tanzania	203.0x	4.765	Human/Stool	Hospital patient	5,134	NCBI:txid622
20	GCA_002949345.1		Tanzania	104.0x	4.613	Human/Stool	Hospital patient	4,597	NCBI:txid622
21	GCA_002949555.1		Tanzania	159.0x	4.613	Human/Stool	Hospital patient	4,597	NCBI:txid622
22	GCA_002949615.1		Tanzania	176.0x	4.613	Human/Stool	Hospital patient	4,597	NCBI:txid622
23	GCA_002949655.1		Tanzania	70.0x	4.49	Human/Stool	Hospital patient	4,597	NCBI:txid622

24	GCA_002949675.1		Tanzania	59.0x	4.578	Human/Stool	Hospital patient	4,863	NCBI:txid622
25	GCA_002949715.1		Tanzania	64.0x	4.708	Human/Stool	Hospital patient	5,061	NCBI:txid622
26	GCA_002949755.1		Tanzania	202.0x	4.452	Human/Stool	Hospital patient	4,752	NCBI:txid622
27	GCA_002949775.1		Tanzania	207.0x	4.450	Human/Stool	Hospital patient	4,703	NCBI:txid622
28	GCA_002949815.1		Tanzania	163.0x	4.647	Human/Stool	Hospital patient	4,968	NCBI:txid622
29	GCA_002949835.1		Tanzania	222.0x	4.562	Human/Stool	Hospital patient	4,892	NCBI:txid622
30	GCA_002949855.1		Tanzania	68.0x	4.596	Human/Stool	Hospital patient	4,881	NCBI:txid622
31	GCA_015475595.1		Germany	170.0x	4.406	Human/Stool	Hospital patient	4,703	NCBI:txid622
32	GCA_000012025.1	*S. boydii*	China	Not provided	4.647	Human/Stool	Hospital patient	4,768	NCBI:txid300268
33	GCA_000020185.1		USA	Not provided	4.875	Human/Stool	Hospital patient	5,129	NCBI:txid344609
34	GCA_001027225.1		South Korea	77.0x	4.574	Human/Stool	Hospital patient	4,666	NCBI:txid621
35	GCA_002949175.1		Tanzania	61.0x	4.726	Human/Stool	Hospital patient	4,956	NCBI:txid621
36	GCA_002949475.1		Tanzania	152.0x	4.887	Human/Stool	Hospital patient	5,083	NCBI:txid621
37	GCA_002949535.1		Tanzania	108.0x	4.703	Human/Stool	Hospital patient	4,925	NCBI:txid621
38	GCA_002950275.1		Tanzania	149.0x	4.802	Human/Stool	Hospital patient	5,062	NCBI:txid621
39	GCA_007106565.1		South Korea	194.0x	4.678	Human/Stool	Hospital patient	4,901	NCBI:txid621
40	GCA_013394535.1		USA	27.896x	4.833	Human/Stool	Hospital patient	5,038	NCBI:txid621
41	GCA_013394555.1		USA	15.67x	4.758	Human/Stool	Hospital patient	4,845	NCBI:txid621
42	GCA_016726285.1		USA	1158.74x	4.549	Human/Stool	Hospital patient	4,679	NCBI:txid621
43	GCA_001518855.1	*S. sonnei*	China	316.02x	5.133	Human/Stool	Hospital patient	5,230	NCBI:txid624
44	GCA_001558295.2		USA	255.63x	4.988	Human/Stool	Hospital patient	5,050	NCBI:txid624
45	GCA_013374815.1		South Korea	167.19x	4.763	Human/Stool	Hospital patient	4,600	NCBI:txid624
